# Nutritional quality of foods and non-alcoholic beverages advertised on Brazilian free-to-air television: a cross-sectional study

**DOI:** 10.1186/s12889-020-08527-6

**Published:** 2020-03-24

**Authors:** Fernanda Helena Marrocos Leite, Laís Amaral Mais, Camila Zancheta Ricardo, Giovanna Calixto Andrade, Julia Soares Guimarães, Rafael Moreira Claro, Ana Clara da Fonseca Leitão Duran, Ana Paula Bortoletto Martins

**Affiliations:** 1 Center for Epidemiological Research in Nutrition and Health (Nupens), University of São Paulo, Avenida Dr. Arnaldo, 715, São Paulo, Brazil; 2 Brazilian Institute for Consumer Defense (Idec), Rua Doutor Costa Júnior, 543, Água Branca, São Paulo, Brazil; 3 Health Sciences Department, University of Ottawa, 3-71 Rue Papineau, Gatineau, Quebec Canada; 4 Nutrition Department, Federal University of Minas Gerais, Avenida Alfredo Balena, 190, Belo Horizonte, Minas Gerais Brazil; 5 Center for Food Studies, University of Campinas, Avenida Albert Einstein, 291, Campinas, São Paulo, Brazil

**Keywords:** Advertising, Television, Foods and beverages, Brazil, Nutrient profiling model

## Abstract

**Background:**

Evidence shows that foods marketed on television are often low-nutrient-dense foods associated with poor nutritional diet quality, obesity and non-communicable diseases. However, little research has been undertaken in Brazil around this issue. This study assessed the nutritional profile of foods and non-alcoholic beverages advertised on Brazilian television by applying the Pan American Health Organization (PAHO) and the World Health Organization (WHO/Europe) nutrient profiling models.

**Methods:**

Cross-sectional study based on the International Network for Food and Obesity/non-communicable diseases Research, Monitoring and Action Support (INFORMAS) protocol. A total of 432 h on the three major Brazilian free-to-air TV channels was recorded from April 1st to 30th 2018. Recordings were done for eight non-consecutive and randomly selected days from 6 am to 12 am (midnight). All food-related ads were coded using a systematic approach and classified according to the PAHO and the WHO/Europe nutrient profile models as “eligible”/“not eligible” for marketing restrictions. Absolute and relative frequencies were used to describe absolute numbers and proportions of food and beverage advertisements. The nutritional profile of foods was compared by day, time of the day and types of TV program. For each advertisement, the parent company of promoted food products, supermarkets and restaurants was identified.

**Results:**

A total of 1610 food and beverage ads were broadcast, representing 18.1% of the total ads shown on selected channels. Over 80.0% of all foods and beverages advertised on Brazilian TV channels did not meet the PAHO and the WHO/Europe nutritional quality standards and were considered eligible for marketing restrictions. The proportion of unhealthy food ads was significantly higher on weekends, in the afternoon, and during soap operas programming. Altogether, 10 transnational and local food and beverage companies, two large international fast food chains and two of Brazil’s largest supermarket retailers accounted for almost 90% of all unhealthy food ads shown.

**Conclusions:**

The findings of the present study indicate a high exposure of the Brazilian population to unhealthy food marketing and an inefficient enforcement of existing regulations. Further research to monitor population exposure to unhealthy food marketing and understand the policy inertia that is preventing policy progress, is highly recommended.

## Background

Prevalence of overweight, obesity and non-communicable diseases (NCDs) has risen substantially in the past three decades in every region, and most rapidly in low- and middle-income countries [1, 2]. This scenario seems to be mainly driven by changes in the global food system, which delivers more processed, energy-dense, palatable, affordable and effectively marketed foods than ever before [3–5]. Changes in dietary patterns with the replacement of unprocessed and minimally processed foods by processed and ultra-processed food products (UPP) have been associated with poor nutritional diet quality [6] (e.g. high in added sugars, sodium, fats, and low in fiber and essential micronutrients) and an increased risk of developing overweight, obesity and NCDs among different age groups [7–12].

Although consumers’ food choices may be influenced by many factors within food environments – including food access, affordability, convenience, food quality and safety, information etc. – the extensive marketing strategies used by the food industry, particularly those directed to children (e.g. persuasive techniques), have been considered one of the main drivers of unhealthy eating behaviors [13]. Reliable evidence indicates that television (TV) food advertising influences food preferences and purchase requests, especially among children and adolescents [14–16]. In addition, studies demonstrate that foods marketed on TV are often UPP which are associated with poor nutritional quality of diets, overweight and obesity [15, 17, 18]. In this context, effective food policies aimed at improving food environments and limiting advertising of unhealthy foods have been highly recommended by international organizations, such as the World Health Organization (WHO) and the Pan American Health Organization (PAHO) [5, 19, 20].

In Brazil, all marketing strategies directed to children are already prohibited by the Consumer Defense Code (*Código de Defesa do Consumidor* - CDC) [21] and reinforced by the National Council of Children and Adolescents’ Rights (*Conselho Nacional dos Direitos da Criança e do Adolescente* - Conanda) Resolution 163/2014 [22]. This legislation covers all kinds of products and services, including food products, but it still lacks appropriate enforcement. This is particularly concerning, given that 60% of Brazilian schoolchildren reported watching TV for more than 2 h/day in 2015 [23]. Considering this scenario, studies that evaluate adherence to current legislation and monitor the exposure of the Brazilian population to unhealthy food marketing (e.g. by day, time of the day, type of advertisers), are still urgently needed. Further, research that determines which foods should be targeted for marketing restrictions and implementation of existing policies in the country is limited.

The aim of this study was therefore to assess the nutritional profile of foods and non-alcoholic beverages advertised on Brazilian TV by applying the PAHO and the WHO Regional Office for Europe (WHO/Europe) nutrient profiling (NP) models. The main hypotheses tested were whether: 1) most food and non-alcoholic beverage advertisements (ads) would not meet the PAHO and the WHO/Europe NP models quality standards; 2) foods and beverages with high critical nutrient content would be most frequently advertised during the weekend and/or audience peak-viewing times (afternoon/evening vs. morning); 3) unhealthy food ads would be mainly promoted by transnational Big Food and Big Soda companies.

## Methods

### Study design

This was a cross-sectional study based on the *Food marketing: television protocol* [24], developed by the INFORMAS, to assess the frequency and level of exposure of different population groups (especially children) to unhealthy food promotion on TV [25]. This global initiative monitors, benchmarks and supports public and private sector actions to promote healthier food environments, reduce obesity and NCDs’ risk factors [13].

### Sample and data collection

In total, 432 h of the three free-to-air channels in Brazil with the highest audience, according to the Kantar-Ibope (Brazilian Institute of Public Opinion and Statistics - IBOPE) [26], were recorded from April 1st to 30th 2018 (*Rede Globo*, *Record* and *SBT* - 144 h/channel). These channels account for 90.5% of the total audience (Globo 47.6%, Record 23.1% and SBT 19.8%) [27]. Recordings were done by a media auditing service for eight non-consecutive and randomly selected days (four weekdays and four weekend days) from 6 am to 12 am (midnight), excluding public holidays and school holiday periods.

All ads were analyzed by 10 trained researchers and one coordinator, and coded in Epi Info™ (version 7.2.2.6) [16] using the INFORMAS protocol [24]. First, an initial screening of the recordings was performed by the researchers, and advertisements were coded as food-related ads – including ads of food or drink products, food or drink companies/brand, and food or drink retailers (e.g. supermarkets, fast food restaurants) - and non-food related ads. During this process, each food-related advertisement received a unique code, while non-food related ads received a generic code. Second, the following information was collected for each advertisement: channel name, date of recording, day of the week or weekend, program name and category, start and end time of the program, time slot of advertisement, advertisement type, company/brand name, name and description of product(s) advertised. The program category was classified into 15 different types, as recommended by the INFORMAS protocol, and grouped into four categories, as follows: soap opera (not specifically for children); news, commentary and political programs; children: cartoon, other show for children; and others (i.e. miscellaneous entertainment, series, movies, reality shows, religious).

When the product advertised consisted of a specific food product (including beverages), coders categorized it - according to the NOVA classification system - into four groups: 1) unprocessed or minimally processed foods; 2) processed culinary ingredients; 3) processed foods; and 4) ultra-processed food and drink products [28]. This classification, proposed by Monteiro et al. [29] was incorporated in the second edition of the Brazilian Dietary Guidelines [30], and is now applied in several countries being recognized as an effective tool for implementing policy actions to promote healthier food environments [31].

Data coding was completed by two trained researchers and any discrepancies were reviewed and resolved by consensus among them, together with the project coordinator. All datasets passed through three crosschecks to eliminate data collection errors; verify start and end time of advertisement (removing any difference greater than three seconds, which was the maximum difference between researchers to pause the recordings during the pre-data-collection test); and check if all ads were coded and rated correctly. The inter-coder reliability was high, ranging from 90.37 to 99.74% before the first crosscheck [24].

### Nutritional quality of foods and beverages advertised

Two NP models were used to assess the nutritional quality of foods and non-alcoholic beverages advertised on Brazilian TV: the PAHO NP model [32] and the WHO/Europe NP model [33]. A summary of the characteristics of both NP models is presented in Table 1.

**Table 1:** Summary of the characteristics of the PAHO and the WHO/Europe NP models

Characteristics	PAHO	WHO/Europe
Application	Restricting marketing of unhealthy foods and beverages to children; regulating school food environments; establishing FOP warning labels, taxation policies and agricultural subsidies; food provision guidelines for social programs.	Restricting the marketing of unhealthy foods and beverages to children.
Categories	Four categories^1^ (based on the extent of industrial processing)	17 categories^2^
Measure	100 g/ml	100 g/ml
Critical nutrients	Sodium, free sugars, non-nutritive sweeteners, total fat, saturated fat, and trans fat.	Energy, salt, non-sugar sweeteners, total fat, saturated fat, total sugar and added sugar.

Note: *FOP* Front-of-package, *PAHO* Pan American Health Organization, *WHO/Europe* WHO Regional Office for Europe

^1^ All food items are classified into four categories, including: 1) ultra-processed products, 2) processed products, 3) unprocessed or minimally processed products, 4) culinary ingredients. Processed or ultra-processed foods are further classified as containing “excessive” or “non excessive” amounts of critical nutrients

^2^ The 17 food categories include: 1) chocolate and sugar confectionery, energy bars, and sweet toppings and desserts; 2) cakes, sweet biscuits, pastries, other sweet bakery wares, and dry mixes for making such; 3) savory snacks; 4) beverages: a) juices, b) milk drinks, c) energy drinks, and d) other beverages; 5) edible ices; 6) breakfast cereals; 7) yogurts, sour milk, cream, and other similar foods; 8) cheese; 9) ready-made and convenience foods and composite dishes; 10) butter and other fats and oils; 11) bread, bread products, and crispbreads; 12) fresh or dried pasta, rice, and grains; 13) fresh and frozen meat, poultry, fish, and similar; 14) processed meat, poultry, fish, and similar; 15) fresh and frozen fruit, vegetables, and legumes; 16) processed fruit, vegetables, and legumes; and 17) sauces, dips, and dressings

The PAHO NP model was chosen because it was specifically developed to be used by the PAHO Member States, including Latin American countries, in a wide range of food and nutrition policies. Foods and beverage items categorized as processed or UPP in the current study adhered to the “excessive” or “non excessive” classification (i.e. free sugar, total fat, saturated fat, trans fat and sodium). The presence of non-nutritive sweeteners in the list of ingredients was also verified. Food products that exceeded at least one of the thresholds for critical nutrients and/or contained any type of non-nutritive sweeteners were considered “eligible” for marketing restrictions [32]. Unprocessed or minimally processed food products and culinary ingredients were not subject to the application of the thresholds and therefore were all considered “not eligible” for marketing restrictions in the present study.

The WHO/Europe NP model was selected for its specific design to restrict marketing of unhealthy foods and beverages to children. Foods and non-alcoholic beverages advertised on Brazilian TV were first classified into one of the 17 possible categories (Table 1), among which the beverages category was further divided into four subcategories (juices, milk drinks, energy drinks and other beverages). Food items classified into five food categories (chocolate and sugar confectionery, energy bars, and sweet toppings and desserts; cakes, sweet biscuits and pastries; other sweet bakery wares, and dry mixes for making such; juices; energy drinks; and edible ices) were automatically considered “eligible” to be restricted, while products classified in two food categories (fresh and frozen meat, poultry, fish and similar; and fresh and frozen fruit, vegetables and legumes) were always considered “not eligible” for marketing restrictions purposes. For foods classified in the remaining food categories (e.g. breakfast cereals; cheeses; butter and other fats and oils etc.), predetermined thresholds of critical ingredients (total fat, saturated fat, total sugar, added sugar, non-nutritive sweetener, salt and energy) per 100 g/ml were used. Food products that exceeded any of the nutrient thresholds established for that specific category were considered “eligible” for marketing restrictions. Additional details about this model can be found in the original source [33].

When an advertisement promoted more than one food or drink product (e.g. supermarket/grocery stores ads), then each product was evaluated separately. For products available in different flavors/types (e.g. whole milk, semi-skimmed milk or skimmed milk), nutritional information was analyzed for all the product presentations shown in the ads as described elsewhere [34]. For restaurant/takeaway ads where combo meals were advertised (e.g. sandwich, sweetened beverages and ice-cream), the following procedures were used: if advertised products were classified in the same food category according to the NOVA classification system, then the entire meal was coded as one item [35]. In this case, the nutritional content of all items was obtained separately and averaged. When there were products from different categories (minimally processed vs. UPP), the analysis was made for each product. Ads of alcoholic beverages (*n* = 156), bottled water (*n* = 2) or any other product not classifiable by the NOVA classification system, such as dietary supplements (*n* = 218), were excluded from the present analyses. A total of 96 food-related ads that did not depict any specific products in the advertisement were further excluded as follows: food brand/company ads (*n* = 21), restaurant or fast food chain ads (*n* = 16) and supermarket or convenience store ads (*n* = 59).

The nutritional content of foods and non-alcoholic beverages advertised was obtained from the nutrition facts label of each product, as described by previous research [36]. This information was captured by photos taken in the five largest supermarket chains in Brazil [37], from April to July 2017. Around 13,000 packaged foods and beverages had all sides of their package photographed according to the methods proposed by Kanter et al. (2017) [38]. Information on name, brand, flavor, package size, nutrition facts and list of ingredients of each packaged product was entered between July and November 2017 by trained nutritionists in an online platform using a template developed by researchers from the Institute of Nutrition and Food Technology (*Instituto de Nutrición y Tecnología de los Alimentos* - INTA), Chile, and the University of North Carolina at Chapel Hill (UNC), United States of America (USA). When that information was unavailable, nutrient data were obtained from company (brand) websites and/or from the Brazilian Table of Food Composition (*Tabela Brasileira de Composição de Alimentos* - TACO) [39]. Nutrient information consisted of energy (in kilocalories), sugar, total fat, saturated fat, carbohydrates (in grams) and sodium (in milligrams). Data were collected per 100 g/ml from all products advertised. Concentrated juices, beverages and dessert instant mixes, powdered milk, teas and coffees, and instant soups were assessed as reconstituted/consumed (100 g/ml) according to the manufacturer’s instructions.

In Brazil, it is not mandatory to declare free sugars on food labels, thus, the amount of this nutrient was estimated by using the method proposed by PAHO, which is based on the information provided on the amount of total sugars [32]. Nevertheless, because it is also not mandatory for food manufacturers to present information on total sugars, only 26.4% of advertised products had this information available on the packaging. Therefore, we estimated the amount of this nutrient for some food products (*n* = 25; 11.6%). Based on the WHO definition of total sugars [33], we considered the total amount of carbohydrates (g) per 100 g/ml declared on food labels as total sugar for milk drinks (*n* = 3), other beverages (i.e. soft drinks, fruit-flavored drinks) (*n* = 11) and yogurts, sour milk, cream and other similar foods (*n* = 11). Finally, the amount of free sugars was estimated by using the method proposed by PAHO [32].

### Data analysis

Absolute and relative frequencies described the number and proportion of food and non-alcoholic beverage ads broadcast on Brazilian TV that would be eligible for marketing restrictions according to the PAHO and the WHO/Europe NP models, as well as the main food and beverage companies/retailers contributing to unhealthy food advertising on the three selected channels. Estimates including weekdays and weekends were weighted to take into account their unequal probability of selection (according to the INFORMAS methodology) [24]. Weights were determined by the inverse probability of selection for weekdays (5.25) and weekend days (2.25) during April 2018 (excluding days associated with religious, national holidays). The nutritional profiles of foods advertised were compared by day (weekday vs. weekend day), time of the day (morning vs. afternoon vs. evening) and types of TV programs. Any difference in the values was considered statistically significant when the 95% confidence interval (CI) did not overlap. Data were analyzed using the statistical software package Stata version 14.2 [40] and weighting factors were incorporated into the analysis (with the command *svy)*.

## Results

### Food and beverage advertising

During the 432 h of recording, 1610 food and non-alcoholic beverage ads were broadcast, representing 18.1% of the total ads shown on the three major free-to-air TV channels in Brazil. The ads featured 216 different food and beverage products from 42 distinct brands, with a mean frequency of 7.5 ads per product and an average rate of 1.2 food ads/channel/hour. UPP, as categorized by the NOVA classification system, constituted the larger proportion of food and beverage ads aired in the selected period (78.9%; 95% CI: 76.7, 81.0) compared to unprocessed/minimally processed foods (15.1%; 95% CI: 13.3, 17.1), processed culinary ingredients (4.3%; 95% CI: 3.3, 5.6) and processed food products (1.6%; 95% CI: 1.1, 2.5) (data not shown).

### Nutrient profile of foods and beverages advertised

Table 2 shows that, as hypothesized in this study, over 80.0% of all foods and beverages advertised on Brazilian TV did not meet either the PAHO or the WHO/Europe nutritional quality standards and were considered eligible for marketing restrictions. The most frequently advertised food categories that exceeded predetermined thresholds for critical nutrients and/or contained non-nutritive sweeteners according to the PAHO NP model were sodas (*n* = 454), processed meats (*n* = 257), convenience foods (*n* = 149), fruit-flavored drinks (*n* = 132), sweetened dairy products (*n* = 77), and candies and desserts (*n* = 72). Similar findings were observed for food ads eligible to be targeted for marketing restrictions according to the WHO/Europe NP model (Table 2). However, although there was consistency between the two models in identifying the major food categories contributing to unhealthy food advertising, the WHO/Europe model eliminated a considerable proportion of food products considered unhealthy by the Brazilian Dietary Guidelines from the group eligible for marketing restrictions, such as convenience foods (14.7%), sweetened dairy products (22.1%) and processed meats (9%), when compared to the PAHO model. On the other hand, food items such as whole milk or sugars were considered eligible for marketing restrictions according the WHO/Europe criteria (due to their high content in fat or sugar), while they were not included in the PAHO NP analysis, since they were classified as unprocessed/minimally processed foods and processed culinary ingredients, respectively, according to the NOVA classification system.

**Table 2:** Number and proportion of food and non-alcoholic beverage ads on Brazilian TV considered eligible for marketing restrictions according to PAHO and WHO/Europe NP models, by food category. April 2018

Food category	Total (n)	Food and beverage ads eligible for marketing restrictions					
		PAHO			WHO/Europe		
		n	%	95% CI	n	%	95% CI
All	1610	1334	80.5	78.4–82.5	1335	80.8	78.6–82.8
Breakfast cereals and granola bars	2	2	100.0	100.0–100.0	2	100.0	100.0–100.0
Bakery products	9	9	100.0	100.0–100.0	9	100.0	100.0–100.0
Convenience foods	149	149	100.0	100.0–100.0	122	81.1	73.5–86.8
Unsweetened dairy products	29	12	40.8	23.7–60.4	20	65.3	44.9–81.3
Sweetened dairy products	77	77	100.0	100.0–100.0	65	85.9	76.0–92.2
Salty snacks	23	23	100.0	100.0–100.0	23	100.0	100.0–100.0
Cookies	53	53	100.0	100.0–100.0	53	100.0	100.0–100.0
Oils and fats	52	41	73.3	58.2–84.3	41	73.3	58.2–84.3
Sauces and dressings	31	28	89.3	71.7–96.5	28	89.3	71.7–96.5
Coffee and tea	43	0	0.0	0.0–0.0	0	0.0	0.0–0.0
Candies and desserts	72	72	100.0	100.0–100.0	72	100.0	100.0–100.0
Cereals, beans, other grain products	17	0	0.0	0.0–0.0	0	0.0	0.0–0.0
Fruits and vegetables	65	0	0.0	0.0–0.0	0	0.0	0.0–0.0
Meat, poultry, seafood, and eggs	77	0	0.0	0.0–0.0	0	0.0	0.0–0.0
Sugar and other noncaloric sweeteners	48	6	11.2	4.9–23.6	48	100.0	100.0–100.0
Processed meats	257	257	100.0	100.0–100.0	249	97.1	94.1–98.6
Juices	2	1	50.0	5.9–94.1	2	100.0	100.0–100.0
Fruit-flavored drinks	132	132	100.0	100.0–100.0	132	100.0	100.0–100.0
Sodas	454	454	100.0	100.0–100.0	454	100.0	100.0–100.0
Cheeses	18	18	100.0	100.0–100.0	15	83.3	59.1–94.5

*CI* confidence interval, *PAHO* Pan American Health Organization, *WHO/Europe* WHO Regional Office for Europe

When analyzing the distribution of TV food advertising over the days of the week and time of the day, we found that the proportion of ads promoting unhealthy food items according to the PAHO and the WHO/Europe NP models was significantly higher on weekends compared to weekdays (91.7%; 95% CI: 89.1, 93.6 vs. 77.8%; 95% CI: 75.2, 80.2; and 91.0%; 95% CI: 88.4, 93.0 vs. 78.3%; 95% CI: 75.7, 80.7, respectively); and in the afternoon compared to the morning or evening viewing times (Table 3). In addition, food and beverage products advertised during soap operas programming had a significantly poorer nutritional profile according to the PAHO criteria when compared to other types of TV programming (Table 3).

**Table 3:** Proportion of food and non-alcoholic beverage ads classified as eligible for marketing restrictions according to the PAHO and the WHO/Europe NP models, by day, time of the day and types of program. April 2018

	Total (n)	Food and beverage ads eligible for marketing restrictions			
		PAHO		WHO/Europe	
		%	95%CI	%	95% CI
*Day*					
Weekday	1023	77.8	75.2–80.2	78.3	75.7–80.7
Weekend	587	91.7	89.1–93.6	91.0	88.4–93.0
*Time of the day*					
Morning	295	77.5	72.0–82.2	74.5	68.9–79.5
Afternoon	603	94.3	92.0–96.0	91.9	89.1–94.0
Evening	712	72.1	68.5–75.4	75.5	72.0–78.7
*Types of program*					
Soap opera (not specifically for children)	423	88.6	85.1–91.3	87.6	84.0–90.4
News, commentary, political programs	315	63.5	57.7–68.9	67.0	61.3–72.3
Children: cartoon, other show for children	172	80.9	73.5–86.5	75.4	67.8–82.0
Miscellaneous entertainment	468	82.1	77.7–85.8	82.6	78.2–86.2
Others^a^	232	84.4	78.4–88.9	86.6	81.0–90.7
**Total**	1610				

*CI* confidence interval, *PAHO* Pan American Health Organization, *WHO/Europe* WHO Regional Office for Europe.

^a^Series, movies, reality shows, religious

### Unhealthy food advertising by advertiser company and product line

The food advertisers with the highest percentage of unhealthy food and beverage ads aired on the three selected channels are presented in Table 4. Altogether, 10 transnational and local food and beverage companies, along with the two largest fast food chains in the world and two of Brazil’s largest supermarket retailers accounted for almost 90% of all unhealthy food ads shown according to both the PAHO (89.9%) and the WHO/Europe (88.5%) NP models, validating our third hypothesis. The Coca-Cola Company and the BRF S.A. occupied the top two positions. Sugary drink brands comprised five out of the top 13 brands most frequently announcing unhealthy food products (data not shown), especially sodas and fruit-flavored drink brands, which were responsible for around 32.0 and 8.0%, respectively, of all ads eligible for marketing restrictions according to both NP models. Ultra-processed meat brands also stood out, accounting for 18.8% of all unhealthy food ads shown on Brazilian TV (data not shown).

**Table 4:** Frequency of the food and beverage companies, fast food restaurants and supermarket retailers contributing to unhealthy (restricted to be marketed by PAHO and the WHO/Europe NP models) food advertising on Brazilian TV. April 2018

Food company	Company type	Main product line	PAHO			WHO/Europe		
			n	%	95%CI	n	%	95% CI
The Coca-Cola Company	Packaged food/beverage	Sodas	266	18.3	16.2–20.5	266	18.2	16.2–20.5
BRF S.A.	Packaged food/beverage	Frozen foods	206	14.3	12.5–16.4	198	13.7	11.9–15.8
3 *Corações* Group	Packaged food/beverage	Coffee drinks	90	4.3	3.5–5.3	90	4.3	3.5–5.3
Burger King Corporation	Fast food	Hamburger	84	7.1	5.7–8.7	84	7.0	5.7–8.7
Dolly, Inc.	Packaged food/beverage	Sodas	76	6.0	4.7–7.5	76	5.9	4.7–7.5
JBS S.A.	Packaged food/beverage	Meat products	75	6.5	5.2–8.1	75	6.5	5.1–8.1
Mondelēz International, Inc.	Packaged food/beverage	Confectionery	71	5.7	4.5–7.3	71	5.7	4.5–7.2
PepsiCo, Inc.	Packaged food/beverage	Sodas, snack foods	63	5.1	4.0–6.6	63	5.1	4.0–6.6
Carrefour S.A.	Supermarket	Diversified	61	5.9	4.7–7.5	72	7.0	5.6–8.7
Nestle S.A.	Packaged food/beverage	Diversified	56	4.2	3.2–5.5	56	4.2	3.2–5.5
McDonald’s Corporation	Fast food	Hamburger	45	3.7	2.8–5.0	25	1.9	1.3–2.9
Yakult Honsha Co., Ltd.	Packaged food/beverage	Sweetened dairy drinks	36	2.7	2.0–3.8	24	1.8	1.1–2.7
Ambev S.A.	Packaged food/beverage	Sodas	35	2.7	2.0–3.8	35	2.7	1.9–3.8
GPA	Supermarket	Diversified	35	3.4	2.5–4.7	46	4.4	3.3–5.8
Others			135	10.0	8.4–11.9	154	11.5	9.8–13.5
**Total**			1334	100.0		1335	100.0	

*CI* confidence interval, *PAHO* Pan American Health Organization, *WHO/Europe* WHO Regional Office for Europe

## Discussion

### Major findings

The present study findings suggest a high exposure of the Brazilian population to unhealthy food marketing. More than 80% of all foods and non-alcoholic beverages advertised on the three most popular free-to-air TV channels were considered eligible for marketing restrictions according to the PAHO and the WHO/Europe NP models, due to their high content of critical nutrients. In addition, most of food and beverage ads shown were of UPP, exceeding six times the number of unprocessed/minimally processed food ads. Considering that 77% of Brazilians (≥16 years) watch TV regularly (seven days/week) and dedicate at least three hours of their day to this activity [41], this frequent exposure to unhealthy food advertising constitutes a particular concern from a public health perspective. Evidence shows that the increasing availability of UPP in the food environment combined with persuasive industry marketing strategies are associated with excessive consumption of these products, leading to an increased risk of developing obesity and NCDs [42]. Besides, there is robust scientific literature demonstrating the role of TV food advertising in shaping food preferences and purchase behaviors among different age groups, particularly children and youth [14, 43, 44].

The predominance of unhealthy food advertising on Brazilian TV has been previously reported by studies conducted in the country over the last 10 years [45, 46], and aligns with research developed according to the INFORMAS protocol in other middle- and high-income countries [47–49]. However, the present study brings an improved understanding of the nutritional quality of food products advertised on Brazilian TV by applying, for the first time, international NP models. Although previous studies carried out in Brazil have also focused their analysis on free-to-air TV channels, they either excluded programs broadcast during the evening hours (from 6 pm to midnight) [46] or analyzed a much smaller sample (*n* = 2732 TV ads), obtained from only two days of TV programming [45]. Additionally, the use of the INFORMAS protocol allowed this paper to extensively categorize all food-related ads according to the NOVA classification system, and to evaluate the distribution of food ads by day and time of the day. This goes beyond of what previous national studies presented.

Consistent with our findings, recent studies carried out in Mexico [50] and New Zealand [35] found that 84 and 69% of food and beverage ads broadcast on free-to-air TV, respectively included at least one food item that did not meet the WHO/Europe NP nutritional quality standards. In Argentina, a survey investigating the nutritional quality of food products advertised on TV between 2013 and 2014 identified that 93% of food and beverage ads did not comply with the PAHO NP model [34].

Based on our sample, sugary drinks (including sodas, sweetened dairy drinks and fruit-flavored drinks), ultra-processed meats, convenience foods, and candies and desserts accounted for the majority of food items containing excessive amounts of critical nutrients according to both NP models. These categories were identified as the most frequently advertised products on TV by both national [45, 46] and international studies [51, 52]. This is also consonant with previous research conducted in Brazil which demonstrated a significant increase in the consumption of UPP, such as sausages, ready meals, sweets, soft drinks and other sugary drinks, between 2002 and 2003 and 2008–2009 [53]. The similarities between the main types of food products advertised and consumed in different countries reinforce existing evidence demonstrating that current food systems are becoming more globalized, industrialized, and dominated by large actors responsible for governing food supply chains, which are increasingly delivering highly palatable, cheap and convenient food products [3, 42, 54, 55].

Although both NP models applied in the present study identified the food categories contributing the most to unhealthy food advertising on Brazilian TV, the results obtained from the PAHO NP model were more aligned with the recommendations presented in the Brazilian Dietary Guidelines [30]. This could be explained by the fact that both the PAHO model and the Brazilian Dietary Guidelines use the NOVA classification system as a basis for identifying less healthy food items by considering their degree of industrial processing. Likewise, the PAHO model was specifically designed to be applied by the PAHO Member States in a wide range of food policies and, for this reason, is considered a more robust criterion for Latin American countries, which are facing a rapid replacement of foods and ingredients used in the preparation of traditional diets by ready-to-eat products [56]. The WHO/Europe NP model, on the other hand, is primarily focused on identifying foods with a high content of critical nutrients excluding, from the group of foods eligible for marketing restrictions in our study, ready-to-eat sandwiches advertised by a fast food retailer that did not exceed the thresholds for fats and salt – even being classified as UPP. At the same time, food items eligible for marketing restrictions by the WHO/Europe model, such as whole milk and table sugars (due to their high content of saturated fat and sugar), would be considered, respectively, as minimally processed food and culinary ingredient, thus, not eligible for marketing restrictions by the PAHO model.

Differences in the nutritional profile of foods and beverages advertised on the three selected channels by day, time of the day and types of programs identified in the current study, demonstrate a significantly higher exposure of the Brazilian population to unhealthy food advertising on weekends, in the afternoon and during soap operas programming. These results can be considered key points of concern for several reasons. First, a survey performed in 2016 found that over half of Brazilians (52%; ≥16 years) reported watching two to five hours of TV on weekends [41]. Second, findings from a recent study carried out in all Brazilian state capitals demonstrated a positive association between the habit of watching TV for more than three hours per day and unhealthy eating behaviors (e.g. higher consumption of sodas and meat products high in fats) among adults (≥18 years) [57]. Third, a higher frequency of unhealthy food and beverage ads in the afternoon period might imply an increased risk of children and adolescents being exposed to them. According to research conducted in Brazil in 2009, which analyzed TV food advertising during children’s programming, the exposure of children to ads promoting “sugars and desserts” over a period of 10 consecutive days was significantly higher in the afternoon compared to the morning [46]. Besides, only one of the three channels selected in the current study has children’s programming [26], which means that children and youth could be exposed to unhealthy food advertising at any time, and particularly during weekends, school breaks or holiday periods. Finally, a literature review on TV food marketing geared towards children in Latin American concluded that the majority of TV advertising was directed to children and their family, and that this exposure was associated with both food preferences and purchases behaviors of families and children being overweight or obese [17].

The limited number of food and beverage manufacturers and food retailers contributing the most to unhealthy food advertising on Brazilian TV is consonant with evidence showing that the global food system is currently controlled by a small number of transnational food and beverage corporations [55, 58, 59]. Our results demonstrate that 10 transnational and local food and beverage companies, two fast food retailers and two of Brazil’s largest supermarket retailers accounted for almost 90% of all unhealthy food ads shown on Brazilian TV. Further, sugary drink companies were responsible for 40% of all unhealthy food ads, followed by ultra-processed meat companies (18.8%). These findings reinforce data presented in previous reports demonstrating that transnational companies, collectively known as Big Food and Big Soda corporations, allocate significant annual budgets to advertising and promotion, including cross-advertising between their brands, to make their products more attractive to consumers and increase their market share, particularly in low- and middle-income countries [60, 61]. Such facts are particularly relevant from a public health perspective and for regulatory purposes, since exposure to unhealthy advertising and promotions can influence consumers’ brand recognition, preferences, purchases and consequently their consumption, especially among children [62].

### Strengths and limitations

This study is central to the current literature since it is the first to investigate the nutritional quality of foods and beverages advertised on Brazilian TV according to the PAHO and the WHO/Europe NP models. It measures, for the first time, the proportion of unhealthy TV advertising by day, time of the day, types of programs and advertising, and it is based on an international methodology (INFORMAS), which allows results to be compared across countries and over time. Additionally, although our study was based on data from April 2018, the sample used included a large number of ads aired on the three major TV channels in Brazil, permitting to identify the main factors contributing to unhealthy marketing on Brazilian free-to-air TV. One limitation was that the present study did not include cable TV channels. However, according to a survey conducted by the Kantar IBOPE media, free-to-air TV is still the main source of information and entertainment in Brazil, reaching 93% of the national population. Also, estimates of advertising expenditure in the country indicate that TV spending represented 70% of total advertising expenditure in 2014 [26].

## Conclusions

This study demonstrates that despite almost two decades of recommendations from national and international organizations, such as WHO and PAHO, the implementation of existent policies to protect children and the general population against unhealthy food advertising on Brazilian TV has been slow and inconsistent. First, eight in 10 food and beverages ads were eligible for marketing restrictions according to the PAHO and the WHO/Europe NP models. Second, around 80% of the ads were for UPP. These figures show that, although the Brazilian Dietary Guidelines presents as two out of its 10 top recommendations “Avoid consumption of UPP” and “Be wary of food advertising and marketing”, Brazilians are exposed daily to TV ads that reinforce the opposite. In such contexts, further research to monitor population exposure to unhealthy food marketing and understand the policy inertia that is preventing policy progress, is highly recommended [42]. Furthermore, in order to put into practice existing advertising restrictions in the country and protect consumers’ rights against any abusive marketing strategies (including unhealthy food advertising), legal practitioners need to be sensitized, well informed and willing to work with public health actors.

One of the barriers that policymakers often need to overcome when implementing policies to reduce a population’s exposure to the advertising of low-nutrient-dense food products is the lack of instruments that help the population differentiate healthier from less healthy foods. According to our findings, the PAHO NP model is more aligned with the Brazilian Dietary Guidelines and overall more restrictive when selecting food items eligible for marketing restrictions compared to the WHO/Europe model. This emphasizes the need to carefully evaluate the characteristics underlying each NP model when developing criteria for use in a specific public health policy [63].

Finally, our study shows that the lack of enforcement of existing regulations to protect consumers’ rights represents a barrier to be overcome to follow the recommendations for a healthy diet presented in the Brazilian Guidelines. Given that the prevalence of obesity has been increasing in Brazil [64] - mainly caused by a shift in dietary patterns towards UPP and sedentary behaviors - and no country has successfully reversed the obesity epidemic [42], complementary policies to enhance synergies and mitigate risks of unhealthy food marketing should be urgently identified and implemented at both national and local levels.

## Data Availability

The INFORMAS “Food Marketing - Television Protocol” is available at: https://ndownloader.figshare.com/files/9885559. Permission to use this protocol has been granted by the INFORMAS’ team to Dr. Ana Paula Bortoletto Martins, who is the Leader of the Program of Health and Sustainable Diets at IDEC (Brazilian Institute for Consumer Defense) and focal point of the INFORMAS in Brazil. The datasets used and analyzed in the current study are available from the corresponding author on reasonable request.

## Abbreviations

*Ads*: Advertisements
*INFORMAS*: International Network for Food and Obesity/Non-communicable diseases Research, Monitoring and Action Support
*NCDs*: Non-communicable diseases
*NP*: Nutrient profiling
*PAHO*: Pan American Health Organization
*TV*: Television
*UPP*: Ultra-processed food products
*WHO*: World Health Organization
*WHO/Europe*: World Health Organization Regional Office for Europe
